# Longitudinal Whole-Exome Sequencing Identifies Clonal Hematopoiesis and Genomic Heterogeneity as a Predictor of Treatment Outcome in Patients with Newly Diagnosed, Elderly Chronic Lymphocytic Leukemia

**DOI:** 10.3390/ijms27062610

**Published:** 2026-03-12

**Authors:** Ho Cheol Jang, Ga-Young Song, Hyeonjin Jeong, Ja Min Byun, Jee Hyun Kong, Myung-won Lee, Won Sik Lee, Ji Hyun Lee, Ho Sup Lee, Ho-Young Yhim, Deok-Hwan Yang

**Affiliations:** 1 Department of Hematology-Oncology, Chonnam National University Hwasun Hospital, Chonnam National University Medical School, 322 Seoyang-ro, Hwasun-eup, Hwasun-gun 58128, Jeollanam-do, Republic of Korea; hd00070@cnuh.com (H.C.J.); songga0e@naver.com (G.-Y.S.); 2 Department of Nursing, Dongshin University, Naju 58245, Jeollanam-do, Republic of Korea; hahajin02@dsu.ac.kr; 3 Department of Internal Medicine, Seoul National University College of Medicine, Seoul National University Hospital, Seoul 03080, Republic of Korea; jaminbyun@snu.ac.kr; 4 Division of Hemato-Oncology, Wonju Severance Christian Hospital, Wonju 26426, Gangwon-do, Republic of Korea; kkongg@yonsei.ac.kr; 5 Division to Hemato-Oncology, Chungnam National University Hospital, Daejeon 35015, Republic of Korea; iyoo23@cnuh.co.kr; 6 Department of Internal Medicine, Hematology, Inje University Busan Paik Hospital, Busan 47392, Republic of Korea; 7 Division of Hematology-Oncology, Department of Internal Medicine, Dong-A University College of Medicine, Busan 49236, Republic of Korea; 8 Department of Internal Medicine, Kosin University College of Medicine, Kosin University Gospel Hospital, Busan 49267, Republic of Korea; hs3667@hanmail.net; 9 Division of Hematology/Oncology, Department of Internal Medicine, Jeonbuk National University Medical School, Jeonju 54896, Republic of Korea; yhimhy@naver.com

**Keywords:** chronic lymphocytic leukemia, whole-exome sequencing, clonal hematopoiesis of indeterminate potential, genomic heterogeneity, chlorambucil–obinutuzumab

## Abstract

Chronic lymphocytic leukemia (CLL) is uncommon in Asia, and longitudinal genomic data from Asian cohorts are limited. We conducted serial whole-exome sequencing (WES) in a multicenter Korean cohort of newly diagnosed, elderly CLL treated with chlorambucil–obinutuzumab to evaluate mutational heterogeneity and clonal hematopoiesis of indeterminate potential (CHIP) during treatment and follow-up. Tumor-only variants were filtered, restricted to nonsynonymous or loss-of-function coding/splice-site mutations, and summarized as a binary patient-by-gene matrix for principal component analysis (PCA), trajectory analysis, and k-means clustering. CHIP was defined as ≥1 qualifying mutation in a prespecified CHIP gene set. Baseline PCA was more compact in patients with complete response at end of treatment, whereas partial response or progressive disease cases were more dispersed. PCA trajectories were compact and directionally consistent in complete responders, more dispersed in partial responders, and highly heterogeneous without a dominant direction in progressive disease. Clustering identified dispersed and compact clusters, and CHIP-associated mutations were enriched in the dispersed cluster (55.6% vs. 8.3%, Fisher’s exact *p* = 0.0086). In paired samples collected 3–5 months after end of treatment, CHIP status changed in some patients. Serial WES may provide complementary information to treatment response, although these observations require confirmation in larger cohorts.

## 1. Introduction

Chronic lymphocytic leukemia (CLL) is the most common adult leukemia in Western populations, yet its incidence is substantially lower in Asian countries [1,2,3]. Despite this epidemiologic difference, the clinical course is similarly heterogeneous, ranging from durable disease control to early relapse or rapid progression [4,5,6]. In Korea, the relative rarity of CLL has limited the availability of cohorts with longitudinal biospecimens and comprehensive genomic profiling, leaving an evidence gap regarding how genomic risk features behave over time in real-world clinical practice.

The therapeutic landscape of CLL has expanded rapidly, including Bruton tyrosine kinase (BTK) inhibitors, BCL2 inhibitor-based regimens, and anti-CD20-containing combinations [7,8,9]. Nonetheless, chemoimmunotherapy backbones such as chlorambucil plus obinutuzumab remain relevant in older or comorbid patients and in settings where treatment selection is constrained [10]. Across treatment strategies, biomarkers that can anticipate long-term outcomes beyond an early response assessment remain clinically valuable for guiding follow-up intensity and the timing of therapeutic adaptation [11].

Whole-exome sequencing (WES) provides a comprehensive view of somatic variation and offers a quantitative framework for summarizing patient-level mutation profiles [12]. Dimensionality-reduction approaches, including principal component analysis (PCA), enable visualization of inter-patient similarities and differences to be potentially used inoperationalizing genomic heterogeneity as mutation-profile patterns [13,14]. In a longitudinal setting, projecting pre-treatment and post-treatment profiles into a shared space further enables within-patient trajectory analysis, potentially revealing evolutionary patterns associated with relapse or resistance [15,16].

Clonal hematopoiesis of indeterminate potential (CHIP) adds a second, distinct dimension to genomic interpretation [17,18]. CHIP is an age-associated expansion of hematopoietic clones that is frequently detected in peripheral blood sequencing performed for hematologic malignancies [17]. Although CHIP can confound attribution of variants to the malignant CLL clone, accumulating evidence suggests that CHIP itself may be associated with treatment tolerance, competing risks, and adverse outcomes [19,20,21]. However, longitudinal studies that jointly evaluate CHIP status and its transitions over time and mutation-profile heterogeneity across treatment timepoints remain limited, particularly in Asian CLL cohorts.

Therefore, this study prospectively assembled a multicenter Korean cohort of newly diagnosed, elderly CLL treated with chlorambucil–obinutuzumab and performed longitudinal WES on samples collected at diagnosis and during follow-up. We focused on two complementary genomic dimensions, specifically mutation-profile heterogeneity quantified from baseline PCA and formalized by unsupervised clustering, and clonal hematopoiesis status and its longitudinal transitions. We then evaluated how these dimensions relate to the treatment completion, response at the end of treatment (EOT), early post-treatment sequencing, and long-term outcomes at the last follow-up.

## 2. Results

### 2.1. Clinical Characteristics and Treatment

**Figure 1:**
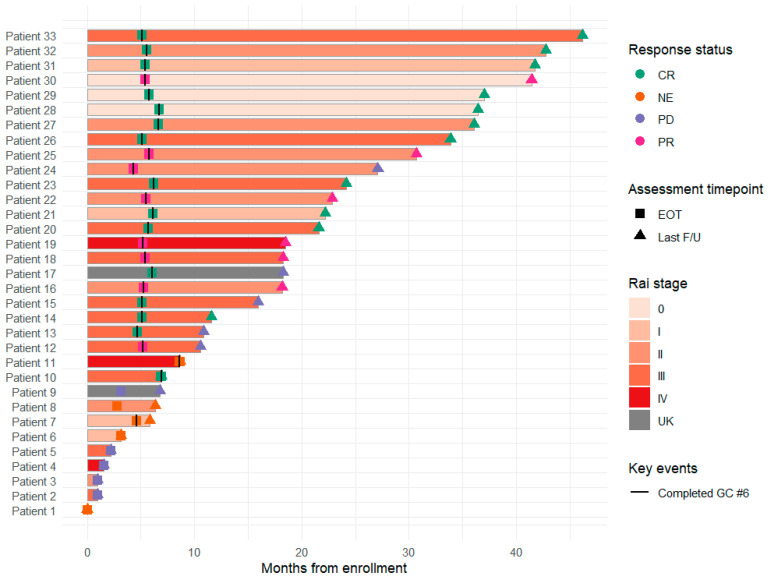
Swimmer plot summarizing treatment course and longitudinal disease assessments in the study cohort. Each horizontal bar represents an individual patient (*n* = 33) from enrollment to the last outpatient follow-up (months). Bar color indicates Rai stage at diagnosis (0–IV; UK, unknown). A black tick mark indicates completion of the planned sixth chlorambucil–obinutuzumab treatment cycle. Disease status is annotated at the end of treatment (EOT; square) and at the last outpatient follow-up (triangle) as complete response (CR), partial response (PR), progressive disease (PD), or not evaluable (NE).

Baseline demographic and clinical characteristics, including conventional karyotype and cytogenetic findings, are summarized in Table 1. Figure 1 summarizes patient-level treatment exposure and follow-up for all 33 enrolled patients. Most patients completed the planned six treatment cycles of chlorambucil–obinutuzumab and underwent end-of-treatment (EOT) response assessment. Response categories were defined as complete response (CR), partial response (PR), progressive disease (PD), and not evaluable (NE). Eight patients discontinued treatment before EOT assessment. Five of these were classified as PD, and three were categorized as NE due to unavailable response assessment. We therefore evaluated whether baseline mutation burden at diagnosis differed between patients who completed therapy (*n* = 25) and those who did not (*n* = 8). The median number of variants was 40 in non-completers and 38 in completers (*p* = 0.7368), and the median number of mutated genes was 31 in non-completers and 32 in completers (*p* = 0.9497) (Wilcoxon rank-sum test). Disease status at the last outpatient follow-up was captured for all patients to reflect longer-term outcomes beyond treatment discontinuation.

### 2.2. Baseline WES Mutation-Profile Heterogeneity and Clinical Status Annotations

**Figure 2:**
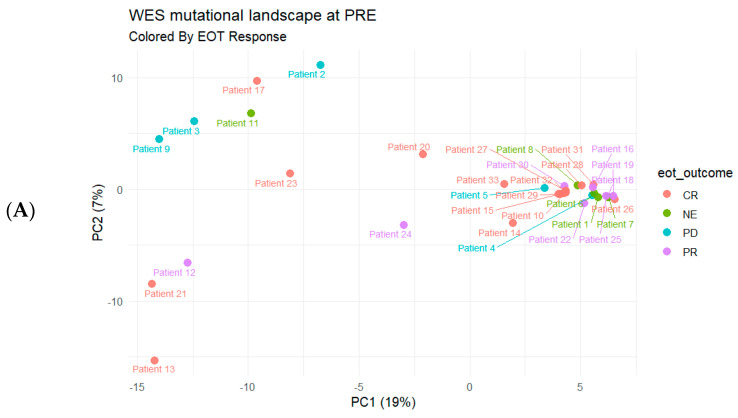

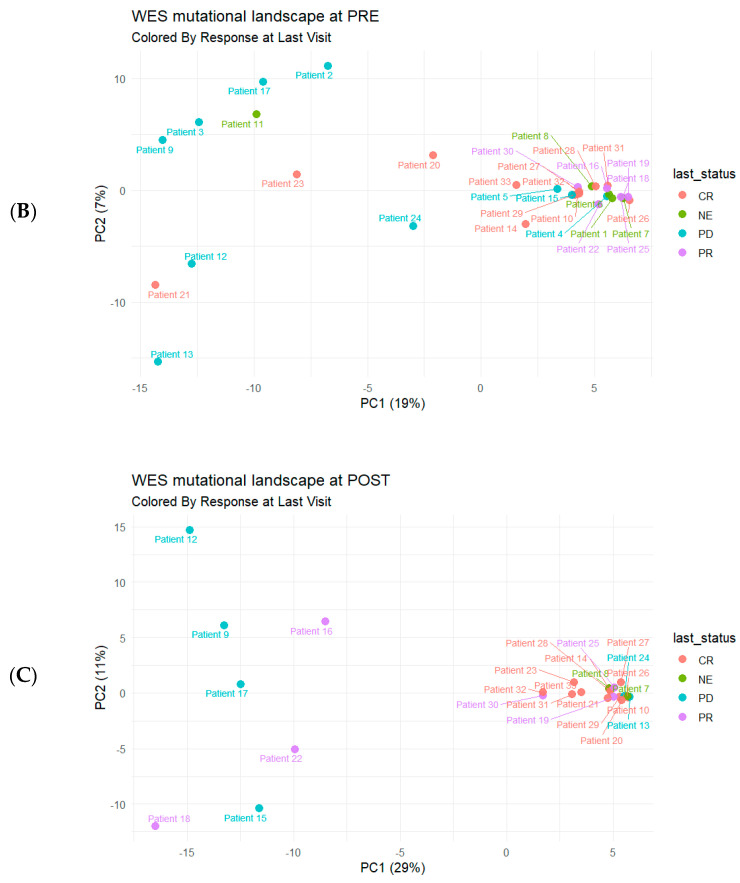
PCA of WES-derived mutation profiles at diagnosis (PRE) and post-treatment (POST). Each point represents one patient, plotted by the first two principal components (percent variance explained shown on the axes). (**A**) PRE samples colored by response category at end of treatment (EOT). (**B**) The same PRE PCA colored by disease status at the last outpatient follow-up. (**C**) POST samples, collected approximately 3–5 months after EOT, shown in a separate PCA and colored by disease status at the last outpatient follow-up. NE indicates not evaluable at the corresponding assessment timepoint.

Baseline WES mutation profiles at diagnosis (PRE) demonstrated inter-patient heterogeneity when summarized by principal component analysis (PCA) (Figure 2). When baseline samples were visualized in PCA space and annotated by EOT response (Figure 2A), patients achieving CR appeared to cluster more compactly, whereas patients with PR or PD were more dispersed across PCA space. A similar pattern was observed when the same baseline profiles were annotated by disease status at the last outpatient follow-up (Figure 2B), with patients showing suboptimal long-term disease control tending to occupy more peripheral regions of the PCA space. Post-treatment WES profiles obtained approximately 3–5 months after EOT (POST) also showed heterogeneous PCA structure (Figure 2C), with broader dispersion among patients with PR or PD at last assessment compared with those with sustained CR.

### 2.3. PRE-to-POST Trajectory Analysis of Mutation-Profile Change During Treatment

**Figure 3:**
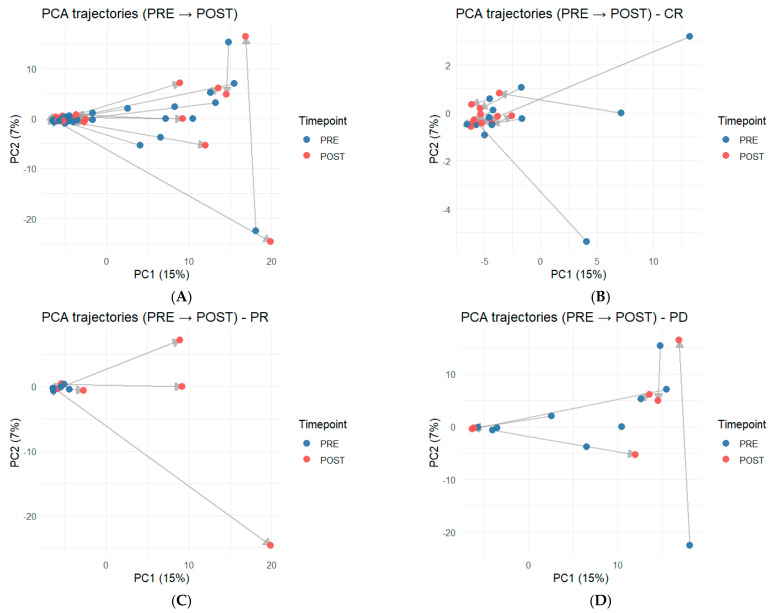
PRE-to-POST PCA trajectories of WES-derived mutation profiles, overall and stratified by response category. Paired PRE and POST samples were projected into a shared PCA space and connected within each patient to visualize trajectories (PRE, blue; POST, red). (**A**) All paired samples. (**B**–**D**) Paired samples stratified by end-of-treatment response category ((**B**) CR; (**C**) PR; (**D**) PD). Variants were filtered using DP ≥ 100 and VAF ≥ 0.05.

Paired PRE- and POST-WES mutation profiles were projected into a shared PCA space and connected to visualize patient-specific trajectories (Figure 3). Across the cohort, trajectories varied in both direction and magnitude (Figure 3A). When stratified by EOT response, patients achieving CR tended to show relatively homogeneous, compact shifts from PRE to POST (Figure 3B). In contrast, PR cases displayed a more dispersed pattern of movement in PCA space (Figure 3C). PD cases exhibited highly heterogeneous trajectories without a consistent dominant direction across patients (Figure 3D). Collectively, these response-associated trajectory patterns indicate that WES-based PCA trajectories can serve as an intuitive summary of longitudinal mutation-profile dynamics during and after treatment.

### 2.4. Baseline Clustering and Enrichment of Clonal Hematopoiesis-Associated Mutations

**Figure 4:**
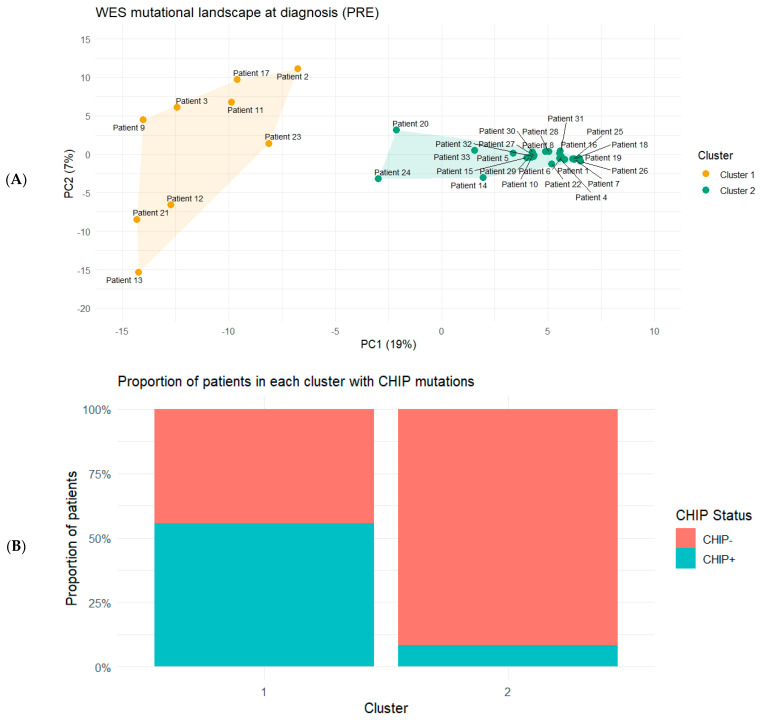
Baseline (PRE) clustering of WES-derived mutation profiles and enrichment of CHIP-associated mutations. (**A**) PCA of PRE samples based on a binary subject-by-gene mutation matrix. Each point represents one patient. K-means clustering (*k* = 2) performed in PCA space identified a dispersed cluster (Cluster 1) and a compact cluster (Cluster 2). (**B**) Proportion of patients in each cluster with at least one CHIP-associated mutation (CHIP+) versus none (CHIP−).

To formalize baseline mutation-profile heterogeneity, pre-treatment WES profiles were summarized as a binary subject-by-gene mutation matrix and clustered in PCA space using k-means (*k* = 2), identifying a dispersed cluster (Cluster 1; *n* = 9) and a compact cluster (Cluster 2; *n* = 24) (Figure 4A). CHIP-associated mutations were more frequent in Cluster 1 than in Cluster 2 (5/9 [55.6%] vs. 2/24 [8.3%]; Fisher’s exact *p* = 0.0086). The per-patient CHIP mutation burden was also high in Cluster 1 (Wilcoxon rank-sum *p* = 0.0040) (Figure 4B). These findings were unchanged in a sensitivity analysis in which CHIP genes were removed from the PCA feature set before clustering, indicating that the cluster separation was not driven solely by CHIP gene mutations.

### 2.5. Longitudinal Transitions of Clonal Hematopoiesis Status and Disease Course

**Figure 5:**
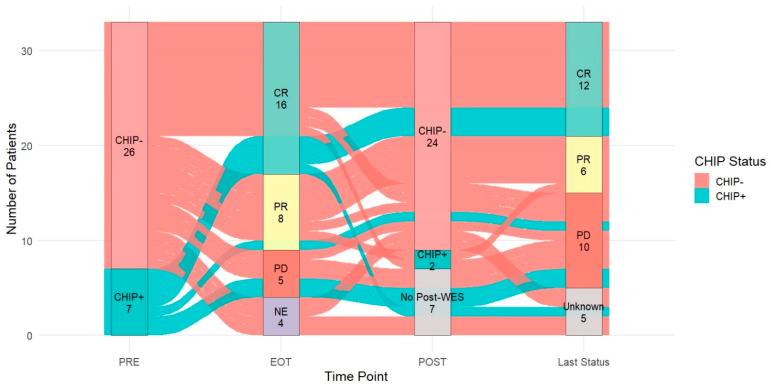
Longitudinal transitions of clonal hematopoiesis status and clinical response across key timepoints. The alluvial plot summarizes patient-level trajectories from diagnosis (PRE), defined by clonal hematopoiesis status (CHIP− or CHIP+), through end-of-treatment (EOT) response, post-treatment WES performed approximately 3–5 months after EOT (POST), and disease status at the last outpatient follow-up (Last Status). Flows connect each patient’s categories across timepoints, highlighting transitions in CHIP status and changes in clinical response. Patients without post-treatment WES are shown as “No Post-WES,” and “Unknown” indicates unavailable last follow-up status.

Figure 5 integrated CHIP status at diagnosis (PRE), response at end of treatment (EOT), 3–5 months after EOT (POST), and disease status at the last outpatient follow-up (Last Status). At diagnosis, 7 patients were CHIP-positive (CHIP+) and 26 patients were CHIP-negative (CHIP−). Four patients were categorized as NE at EOT because they did not reach the EOT assessment. Post-treatment WES was available for 26 patients (CHIP−, *n* = 24; CHIP+, *n* = 2), while 7 patients had no post-treatment WES. Among patients with paired PRE and POST sequencing, CHIP status changed over time, including both loss of baseline CHIP positivity and emergence of post-treatment CHIP positivity. Collectively, the alluvial plot provides an integrated, patient-level view of how baseline host clonal architecture (CHIP status) aligns with treatment response, availability, and results of early post-treatment genomic assessment, and longer-term clinical status, highlighting that clinical outcomes during follow-up can arise from multiple distinct molecular and treatment course pathways rather than a single uniform trajectory.

## 3. Discussion

In this longitudinal cohort of newly diagnosed CLL treated with chlorambucil–obinutuzumab, we used serial WES to characterize baseline mutation-profile heterogeneity and CHIP across diagnosis, treatment, early post-treatment assessment, and long-term outpatient follow-up. Because CLL is relatively uncommon in Asian populations, longitudinal genomics datasets from Asian cohorts remain limited. In this context, the availability of paired clinical trajectories and serial sequencing represents a practical strength of the present study.

A key clinical observation from the treatment timelines was that response assessment at the end of treatment, while clinically meaningful, did not fully reflect long-term disease status for all patients. Several individuals who achieved CR at EOT subsequently had PR, PD, or non-CR status at the last follow-up. When interpreted alongside the baseline PCA results, these discordant courses were more often observed among patients whose baseline mutation profiles were more dispersed in PCA space. Although this PCA-based description is not a substitute for validated prognostic modeling, it supports the concept that baseline genomic heterogeneity may help identify patients who warrant closer surveillance even after an apparent CR.

Patients who discontinue therapy early are often expected to harbor a higher baseline mutation burden, consistent with a more aggressive disease course. In our cohort, however, baseline mutation burden at diagnosis did not differ between patients who completed treatment and those who did not. Notably, most non-completers experienced PD, indicating that early clinical deterioration can occur even in the absence of a higher overall mutation count. This suggests that prognosis may be influenced by factors not captured by simple burden metrics alone. In this context, the dispersion observed in PCA supports the concept that pattern-level genomic heterogeneity provides information complementary to mutation burden and may better reflect biologically meaningful differences relevant to response heterogeneity and follow-up status.

Trajectory analysis extended the use of WES beyond static baseline profiling by projecting paired PRE and POST samples into a shared PCA space. Within this framework, we observed the response-associated differences in the magnitude and direction of within-patient movement, with more compact shifts among CR cases and more variable, sometimes larger displacements among PR or PD cases. These findings suggested that WES-based movement in mutation-profile space may serve as an intuitive summary of longitudinal molecular dynamics during and after therapy. Importantly, this is an exploratory representation rather than a definitive mechanistic readout, and it should be tested and calibrated in larger cohorts with harmonized sampling timepoints and outcome definitions.

CHIP provided an additional and conceptually distinct dimension. CHIP is often treated primarily as a confounder in tumor-only sequencing of peripheral blood or bone marrow because detected variants can arise from non-malignant hematopoietic clones. In our cohort, however, CHIP-associated mutations were enriched among patients in the dispersed baseline cluster and were accompanied by a higher per-patient CHIP mutation burden. These results suggest that CHIP status may function not only as a technical consideration for variant attribution, but also as a host-related feature that co-segregates with broad molecular heterogeneity. On the other hand, CHIP was not determinative at the individual level. Some of the CHIP-positive patients could complete the therapy and achieved a CR, underscoring that CHIP should be interpreted as a risk-enriching characteristic rather than a binary predictor of outcome.

From a clinical implementation perspective, WES is not routinely performed for CLL in many settings, particularly after treatment completion. Nevertheless, our longitudinal data provide a rationale for considering post-treatment genomic reassessment in selected patients, particularly those with more heterogeneous baseline mutation profiles or evidence of CHIP-associated mutations at baseline. We also observed that CHIP status was not uniform over time, with both loss of baseline CHIP positivity and emergence of post-treatment CHIP positivity observed among patients with paired PRE-to-POST sequencing. While the clinical meaning of these transitions could not be established definitively in this modest cohort, the observation supports the hypothesis that post-treatment sampling may reveal evolving host clonal architecture that is not evident from EOT response alone.

The dynamic nature of CHIP across peripheral blood and bone marrow raises mechanistic questions that cannot be resolved by tumor-only WES. Apparent disappearance or emergence of CHIP-associated variants may reflect therapy-related selection, clonal competition, differences in sample cellular composition, sampling variability, or compartment-specific biology. Future studies integrating orthogonal validation and improved clonal resolution, including matched germline sequencing, cell fractionation or CD19-enrichment, and deeper targeted sequencing will be important for separating malignant CLL-derived variants from CHIP-derived variants and for quantifying smaller clones below standard WES sensitivity thresholds.

Several limitations should be acknowledged. The cohort size is modest and the sample availability varied across timepoints, which may introduce selection and survivorship biases. Tumor-only WES from mixed-cell samples limited a clonal attribution and the CHIP gene list overlapped with the genes that can be altered in CLL, which could complicate interpretation. In addition, PCA and k-means-based summaries could be sensitive to feature selection and missingness, and the present findings should be viewed as hypothesis-generating rather than confirmatory. Finally, because we did not include an external Western cohort analyzed using a fully harmonized pipeline, we could not perform a formal cross-population comparison and therefore cannot determine whether the genomic patterns observed here are population-specific. Prospective validation in larger cohorts and under contemporary treatment regimens, including BTK inhibitor or venetoclax-based therapies, will be required before clinical translation.

In conclusion, serial WES could provide additional information to the results of EOT response with baseline mutation-profile heterogeneity and dynamic transitions of CHIP status in newly diagnosed, elderly CLL treated with chlorambucil–obinutuzumab. The baseline dispersion in PCA space, response-associated PRE-to-POST trajectory patterns, enrichment of CHIP in the dispersed cluster, and observed longitudinal CHIP transitions could collectively support further evaluation of WES-informed risk stratification and surveillance strategies in CLL.

## 4. Material and Methods

### 4.1. Study Design and Patients

This multicenter, prospective, longitudinal study included 33 patients with newly diagnosed, elderly CLL treated with chlorambucil–obinutuzumab. Peripheral blood or bone marrow aspirates were collected at diagnosis (PRE) and approximately 3 to 5 months after end of treatment (POST). Clinical information was abstracted from medical records, including Rai stage, laboratory results including complete blood count (CBC) and lactate dehydrogenase (LDH), response assessments, and disease status at end of treatment (EOT) and the last outpatient follow-up. Response and disease status were categorized as complete response (CR), partial response (PR), progressive disease (PD), or not evaluable (NE) at EOT and at the last outpatient follow-up. Response was assessed according to the International Workshop on Chronic Lymphocytic Leukemia (iwCLL) criteria. This study was conducted in accordance with institutional review requirements and the Declaration of Helsinki. Written informed consent was obtained from all participants.

### 4.2. Sample Collection, Whole-Exome Sequencing, and Primary Processing

Genomic DNA was extracted from EDTA whole blood or bone marrow aspirates using the Chemagic Magnetic Separation Module I method with the DNA Blood 20 µL kit (PerkinElmer chemagen Technologie GmbH, Baesweiler, Germany). Libraries were prepared using MGIEasy Exome Capture V5 (MGI Tech Co., Ltd., Shenzhen, China) and sequenced on the MGI DNBSEQ-G400 platform. Whole-exome sequencing reads were aligned to the GRCh38/hg38 (Genome Reference Consortium Human Build 38) reference genome using BWA-MEM (Burrows–Wheeler Aligner—Maximum Exact Matches, ver0.7.17), converted to coordinate-sorted BAM (Binary Alignment/Map) format with SAMtools, and duplicate reads were marked using Picard. Base quality score recalibration was performed with Genome Analysis Toolkit (GATK) BaseRecalibrator/ApplyBQSR using a Genome Aggregation Database (gnomAD) allele frequency resource as known sites [22]. Somatic variant calling was performed in a tumor-only mode with GATK Mutect2 [23], restricted to exome target intervals, followed by GATK FilterMutectCalls to generate filtered VCF (Variant Call Format) files. From filtered VCFs, variants passing all Mutect2 filters were retained using GATK SelectVariants. Variant-level sequencing depth (DP) and allele depths (ADs) were extracted, and variant allele fraction (VAF) was calculated as alternate-allele reads divided by total reads using VCF genotype fields. These VCF-derived DP and VAF metrics were used for downstream filtering and visualization. Passing variants were annotated using ANNOVAR (2024Oct08) to generate multi-annotation tables [24]. For downstream analyses, variants were restricted to autosomes, sex chromosomes, and mitochondrial DNA, limited to exonic or splicing regions, and focused on nonsynonymous or loss-of-function consequences. High-confidence variants were defined using VCF-derived thresholds of DP ≥ 100 and VAF ≥ 0.05.

### 4.3. Gene-Level Mutation Profiling, PCA, and Baseline Clustering

A gene was considered mutated in a sample if at least one qualifying variant was present in that gene. A binary subject-by-gene matrix was constructed at diagnosis (PRE), and genes observed in fewer than two subjects were excluded from the principal component analysis (PCA) feature space to stabilize dimension reduction [25]. PCA was performed on the centered and scaled binary matrix, unsupervised k-means clustering was performed in PCA space. PCA plots were generated with points labeled by a consistent patient index and colored by EOT response or last follow-up disease status. To evaluate temporal shifts in mutational profiles, PRE and POST samples were combined into a unified subject–timepoint matrix. When duplicate samples existed for a given subject and timepoint, the sample retaining the largest number of post-filter variants was selected. PRE-to-POST trajectories were visualized as arrows in PCA space overall and stratified by response category.

### 4.4. Clonal Hematopoiesis Definition and Statistical Testing

CHIP status was defined at the sample level by the presence of ≥1 qualifying mutation in a prespecified CHIP gene set including DNMT3A, TET2, ASXL1, PPM1D, TP53, JAK2, spliceosome genes, and related canonical CHIP genes, using the same DP, VAF, and functional filters applied to the WES call set [17,18]. CHIP prevalence and per-patient CHIP mutation burden were compared between clusters and across timepoints using Fisher’s exact tests and Wilcoxon rank-sum tests. Permutation testing was used as an additional robustness check for set-level burden differences.

### 4.5. Clinical Outcomes and Disease-State Transitions

Swimmer plots summarized follow-up duration from enrollment, incorporating Rai stage at baseline, completion of the 6th treatment cycle, and response assessments at EOT and at last follow-up. Laboratory trends including absolute neutrophil count (ANC), absolute lymphocyte count (ALC), platelet count (PLT), and LDH were visualized longitudinally by standardized treatment cycle indexing. To summarize categorical transitions across the study timeline, an alluvial plot integrated diagnosis CHIP status, treatment response, and last follow-up disease status.

### 4.6. Software and Statistics

Primary processing used BWA-MEM (ver0.7.17), SAMtools (ver1.23), Picard (ver3.4.0), and GATK (ver4.5.0.0). Variant annotation used ANNOVAR. Statistical analyses and figure generation were performed in R (ver 4.3.2), including vcfR for VCF parsing [26], ggplot2 for PCA visualizations, ggalluvial for alluvial plots, and standard hypothesis tests with multiple-testing correction. Unless otherwise stated, two-sided *p* < 0.05 was considered statistically significant.

## 5. Conclusions

In this longitudinal cohort of newly diagnosed, elderly chronic lymphocytic leukemia was treated with chlorambucil–obinutuzumab, serial whole-exome sequencing enabled characterization of inter-patient genomic heterogeneity at diagnosis, and dynamic changes in CHIP-associated variants across timepoints were present. Baseline mutation burden did not explain early treatment discontinuation, whereas unsupervised, pattern-level summaries of the mutational landscape captured heterogeneity that aligned with response categories and longer-term follow-up status. CHIP-associated variants were enriched within the dispersed baseline mutational pattern, highlighting the importance of considering host clonal hematopoiesis when interpreting serial blood or marrow sequencing in older patients. Although these findings are exploratory and limited by a modest sample size, they support further evaluation of longitudinal whole-exome sequencing as a complementary approach to conventional response assessment for risk enrichment and post-treatment monitoring in chronic lymphocytic leukemia. Larger studies with longer follow-up will be required to validate clinical utility and clarify the biological basis of the observed mutational patterns.

## Figures and Tables

**Table 1:** Baseline demographic, clinical, and cytogenetic characteristics of the study cohort. Continuous variables are presented as median (interquartile range, IQR), and categorical variables are presented as number (percentage). “Unknown” indicates missing or unavailable test results. ECOG, Eastern Cooperative Oncology Group, IGHV, immunoglobulin heavy chain variable region.

Characteristic	Value
Age, years	77 (IQR 72–80)
Sex	
Male	19 (57.6%)
Female	14 (42.4%)
ECOG performance status	
0	6 (18.2%)
1	25 (75.8%)
2	2 (6.1%)
Rai stage	
0	3 (9.1%)
I	5 (15.2%)
II	8 (24.2%)
III	12 (36.4%)
IV	3 (9.1%)
Unknown	2 (6.1%)
Binet stage	
A	8 (24.2%)
B	8 (24.2%)
C	15 (45.5%)
Unknown	2 (6.1%)
Lymphadenopathy	
Yes	14 (42.4%)
No	17 (51.5%)
Unknown	2 (6.1%)
Splenomegaly	
Yes	13 (39.4%)
No	18 (54.5%)
Unknown	2 (6.1%)
Hepatomegaly	
Yes	2 (6.1%)
No	30 (90.9%)
Unknown	1 (3.0%)
IGHV status	
Yes	1 (3.0%)
No	13 (39.4%)
Unknown	19 (57.6%)
Conventional karyotype	
Normal	27 (81.8%)
Abnormal	6 (18.2%)
del (13q)	
Positive	5 (15.2%)
Negative	27 (81.8%)
Unknown	1 (3.0%)
del (17p)	
Positive	0 (0.0%)
Negative	32 (97.0%)
Unknown	1 (3.0%)
del (11q)	
Positive	3 (9.1%)
Negative	29 (87.9%)
Unknown	1 (3.0%)
Trisomy 12	
Positive	0 (0.0%)
Negative	32 (97.0%)
Unknown	1 (3.0%)

## Data Availability

All data supporting the findings of this study are included in this article. No additional DNA sequencing datasets were generated beyond the assays described in the Section 4. The analysis workflow is described in Section 4. Further clarifications are available from the corresponding author upon reasonable request.
